# Brightly Luminescent and Moisture Tolerant Phenyl Viologen Lead Iodide Perovskites for Light Emission Applications

**DOI:** 10.1021/acs.jpclett.1c01271

**Published:** 2021-06-03

**Authors:** Elena Blundo, Antonio Polimeni, Daniele Meggiolaro, Andrea D’Annibale, Lorenza Romagnoli, Marco Felici, Alessandro Latini

**Affiliations:** † Dipartimento di Fisica, Sapienza Università di Roma, Piazzale Aldo Moro 5, 00185 Roma, Italy; ‡ Computational Laboratory for Hybrid/Organic Photovoltaics (CLHYO), Istituto CNR di Scienze e Tecnologie Chimiche “Giulio Natta” (CNR-SCITEC) Via Elce di Sotto 8, 06123 Perugia, Italy; § Dipartimento di Chimica, Sapienza Università di Roma, Piazzale Aldo Moro 5, 00185 Roma, Italy

## Abstract

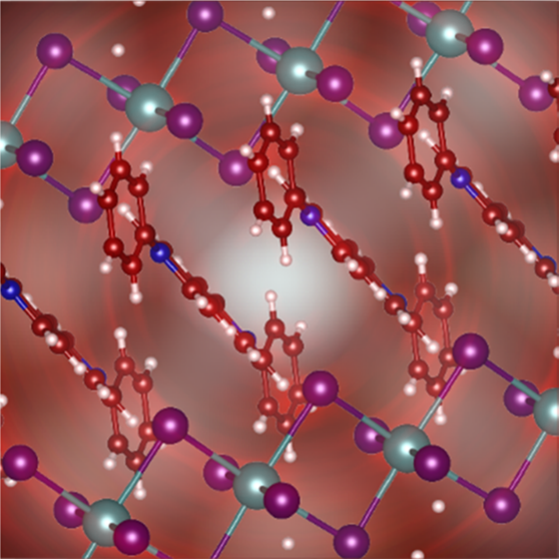

Lead halide perovskites are outstanding materials for optoelectronics, but they typically feature low stability against external agents. To overcome this drawback, LHPs based on quaternary ammonium cations, such as phenyl viologen lead iodide (PhVPI), were found to be promising candidates, being water-resistant and thermally stable. In this Letter, the optoelectronic properties of the PhVPI are investigated by a combined experimental–theoretical approach. Although the as-prepared material is photoluminescence-inactive, a short thermal (5 min @ 290 °C) or laser annealing turns PhVPI into a highly luminescent material, in the 600–1000 nm range. The PhVPI PL emission was characterized at different annealing conditions, and the structural evolution following thermal treatments was investigated by means of X-ray diffraction, Raman, and NMR spectroscopies. Besides this, the electronic structure and emission properties were investigated by density functional theory simulations. The intense optical emission and high stability make PhVPI an intriguing material for applications related to light-emitting devices.

Lead halide perovskites (LHPs) are intensely investigated because of interesting optoelectronic properties,^1^ such as the direct band gap^2,3^ and large absorption coefficients, ease of synthesis, versatile chemistry, and solution processability.^4−6^ These properties were exploited for solar cells, achieving conversion efficiencies as high as 25.5%,^7^ which are mainly related to the long lifetimes of photogenerated carriers^8,9^ and the high defect tolerance.^10−12^ LHPs also attracted attention for the development of efficient light-emitting devices.^13,14^ However, they suffer from a poor chemical and thermal stability. In addition to intrinsic instabilities related to the expulsion of iodine and the formation of metal lead under light irradiation,^15−18^ the Brønsted acidity of methylammonium is responsible for the limited thermal stability and for the extreme sensitivity to atmospheric moisture of methylammonium lead iodide perovskites (MAPI).^19^ Intense efforts have thus been devoted to improve the long-term stability of LHPs. Mixing organic with inorganic cations, e.g., Cs,^20^ and using mixed 3*D*/2D perovskites to control oxygen and water diffusion in the active layer^21^ are valuable strategies.

An alternative approach lies in the use of quaternary ammonium cations, which do not possess acidic protons and consequently eliminate the problem of the Brønsted acidity-related instability. The validity of this approach was demonstrated for tetramethylammonium lead iodide, the simplest quaternary ammonium iodoplumbate (TMPI).^22^ TMPI was found to be more thermodynamically stable than MAPI and completely insensitive to water under ambient conditions.^23^ Considering the extent of the quaternary ammonium cation class, many materials with interesting optoelectronic properties may be designed, with the advantage of increasing the electronic coupling of the inorganic/organic moieties, particularly when highly conjugated organic cations are used.

This is especially true in the case of viologen cations.^24−26^ Viologen-based perovskites possess interesting electronic properties, stemming from charge transfer processes between the inorganic Pb–I framework and the electron acceptor cations.^27^ Among them, phenyl viologen lead iodide (PhVPI) was recently synthesized, demonstrating remarkable thermal stability, insensitivity to water at ambient temperature and an optical band gap of 1.34 eV, lower than methyl viologen lead iodide (2.0 eV) due to phenyl viologen having a more extended conjugation.^28^ These properties, coupled to the easy synthesis and solution processability (it is soluble in aprotic polar solvents, such as DMF) make PhVPI a valuable candidate for optoelectronic applications.

To evaluate the potentiality of viologen perovskites for optoelectronics, a detailed understanding of their electronic and optical properties and the mechanism underlying the charge transfer processes is required.

In this work a combined experimental-computational analysis of the optoelectronic properties of PhVPI—as prototype of the phenyl viologen perovskite class—is carried out. First, the electronic and optical properties of PhVPI are investigated by density functional theory (DFT) and many body perturbation theory (MBPT) calculations. Then, the photoluminescence (PL) properties of PhVPI and the effects of laser and thermal annealing are discussed. Our study shows that while the as-prepared PhVPI is totally PL-inactive, its emission increases dramatically upon laser-annealing or thermal annealing at 290 °C for 5 min, a temperature slightly below its decomposition point. Optical activation through annealing renders PhVPI an efficient light-emitter, whose emission is also remarkably stable vs temperature. Structural changes occurring upon annealing are investigated by X-ray diffraction (XRD), nuclear magnetic resonance (NMR) and Raman spectroscopies. The origin of the intense emission and its stability vs *T* are discussed based on a DFT analysis.

The crystal structure of PhVPI is characterized by 1D Pb–I channels of the inorganic alternated to columns of stacked phenyl viologen cations; see Figure 1a,b. The band structure and the projected density of states over atomic orbitals (PDOS) of the material, calculated by using the PBE functional^29^ and including spin–orbit coupling (SOC), are shown in Figure 1c. At the PBE-SOC level of theory, PhVPI shows a nearly direct band gap of 0.58 eV near the Γ point of the Brillouin zone (BZ). The top of the valence band (VB) mainly arises from I-p orbitals, while the conduction band (CB) is mainly associated with localized molecular orbitals of the organic cation. A more accurate estimate of the electronic band gap is obtained by G_0_W_0_ calculations, SOC included (see Computational details), predicting a gap of 2.02 eV close to the Γ point.

**Figure 1:**
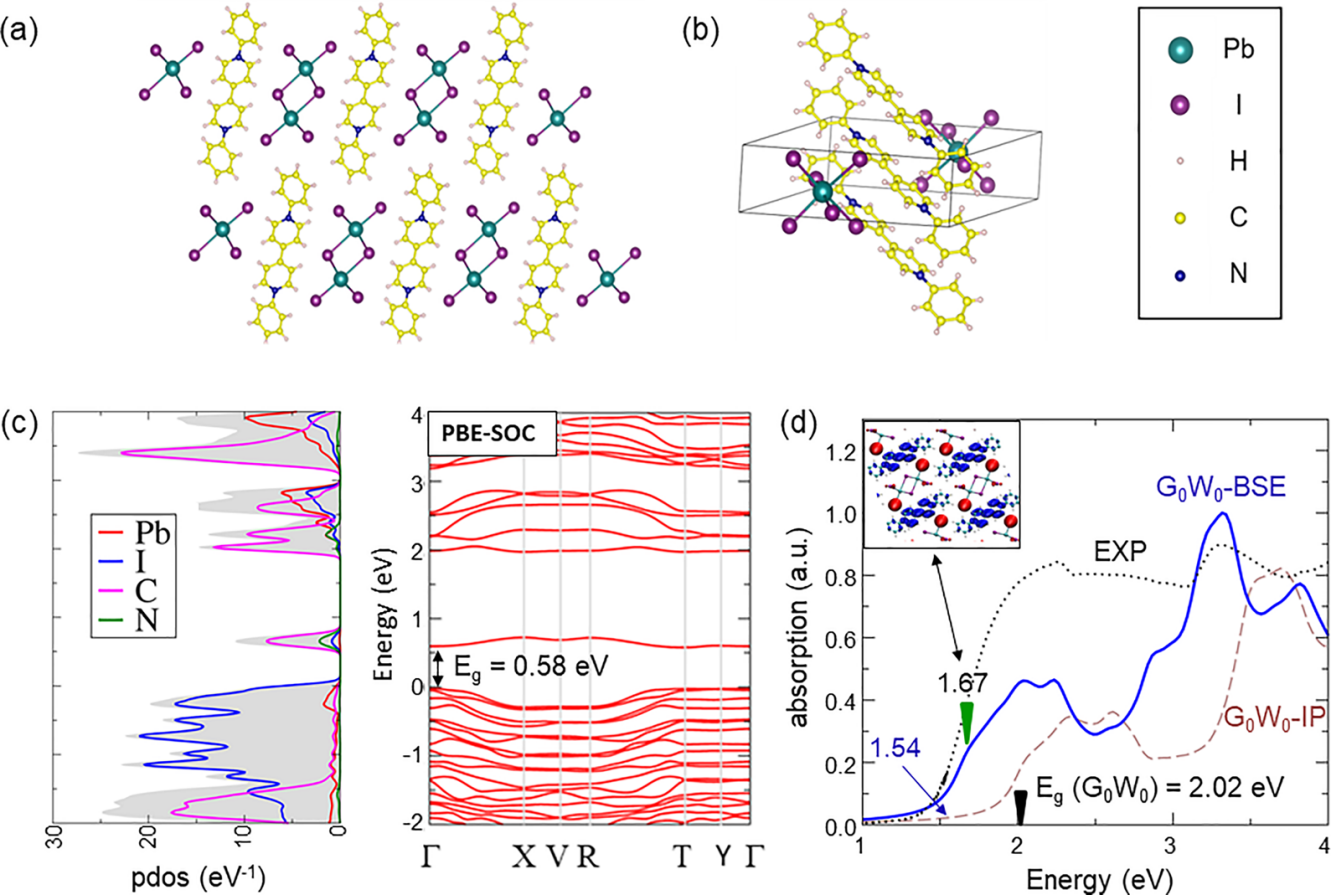
PhVPI crystal structure (a) top and (b) side views. (c) PDOS and electronic band structure calculated at the PBE-SOC level. (d) Calculated absorption spectra at the G_0_W_0_-SOC independent particles level of theory (G_0_W_0_-IP brown line) and by including exciton effects (G_0_W_0_-BSE blue line), compared with the room-temperature experimental absorption spectrum (black points). Inset: orbitals associated with the optical transition at 1.67 eV, i.e., the first intense transition close to the absorption edge.

The optical spectrum of PhVPI was simulated by solving the Bethe–Salpeter equation (BSE) on top of G_0_W_0_-SOC calculations. In Figure 1d, the calculated G_0_W_0_ independent particles (G_0_W_0_-IP), G_0_W_0_-BSE and experimental absorption spectra are compared. In the G_0_W_0_-IP optical spectrum, the absorption edge occurs at the G_0_W_0_ electronic gap (2.02 eV), while the inclusion of exciton effects through the solution of the BSE (G_0_W_0_-BSE) leads to a down-shift of the absorption edge to 1.54 eV, indicating the presence of strongly bound excitons with binding energies up to ∼0.5 eV. The optical spectrum is characterized by a broad absorption band from 1.54 to ∼2.5 eV and a more intense band between 2.5 and 4.0 eV.

The broad absorption band in the low energy range (<2.5 eV) is the convolution of several exciton transitions associated with complex charge transfer between the inorganic I-p orbitals in the VB and the lowest unoccupied molecular orbital (LUMO) of the organic cation in the CB, in agreement with the PDOS and previous studies on methyl viologen compounds.^27^ The calculated G_0_W_0_-BSE optical spectrum reproduces fairly well the absorption threshold and the main optical features of the experimental spectrum. Discrepancies in the intensities are ascribed to the anisotropy of the system and the thermal motion of the organic cations at room-temperature, modulating the electronic states at the band edges. Notably, the electronic and optical properties of PhVPI are profoundly different from 3D LHPs, *e.g*., MAPI, for which both VB and CB arise from the Pb–I inorganics with no contribution of the methylammonium cation to the band edges,^30^ and lower exciton binding energies (a few tens of millielectronvolts) are reported.^9,31^ The considerable exciton binding energy estimated by BSE calculations suggests that PhVPI and related materials may be exploited in the development of light emitting devices.

To assess this possibility, the emission properties of polycrystalline PhVPI were investigated by PL spectroscopy. PhVPI was synthesized as in ref (28). PL experiments were performed with a 532.2 nm excitation laser; see Supporting Information. As shown in Figure 2a, the as-prepared sample results to be PL-inactive. However, by increasing the laser power, slight surface color changes can be noticed, and concomitantly, a PL signal arises. Relatively modest laser powers—focused via a 20× objective for a few seconds—are sufficient to observe such an effect; see Figure 2a. After laser-annealing the sample for 6 s with laser power *P*_ann_ = 200 μW, we reacquired the spectrum with lower excitation power *P*_exc_ = 50 μW, and a weak luminescence could be observed (blue spectrum). A much higher PL efficiency—comparable to that of high-quality InP epilayers—is achieved by increasing the annealing power P_ann_ to 600 μW and using a 1 s annealing time; see Figure 2b.

**Figure 2:**
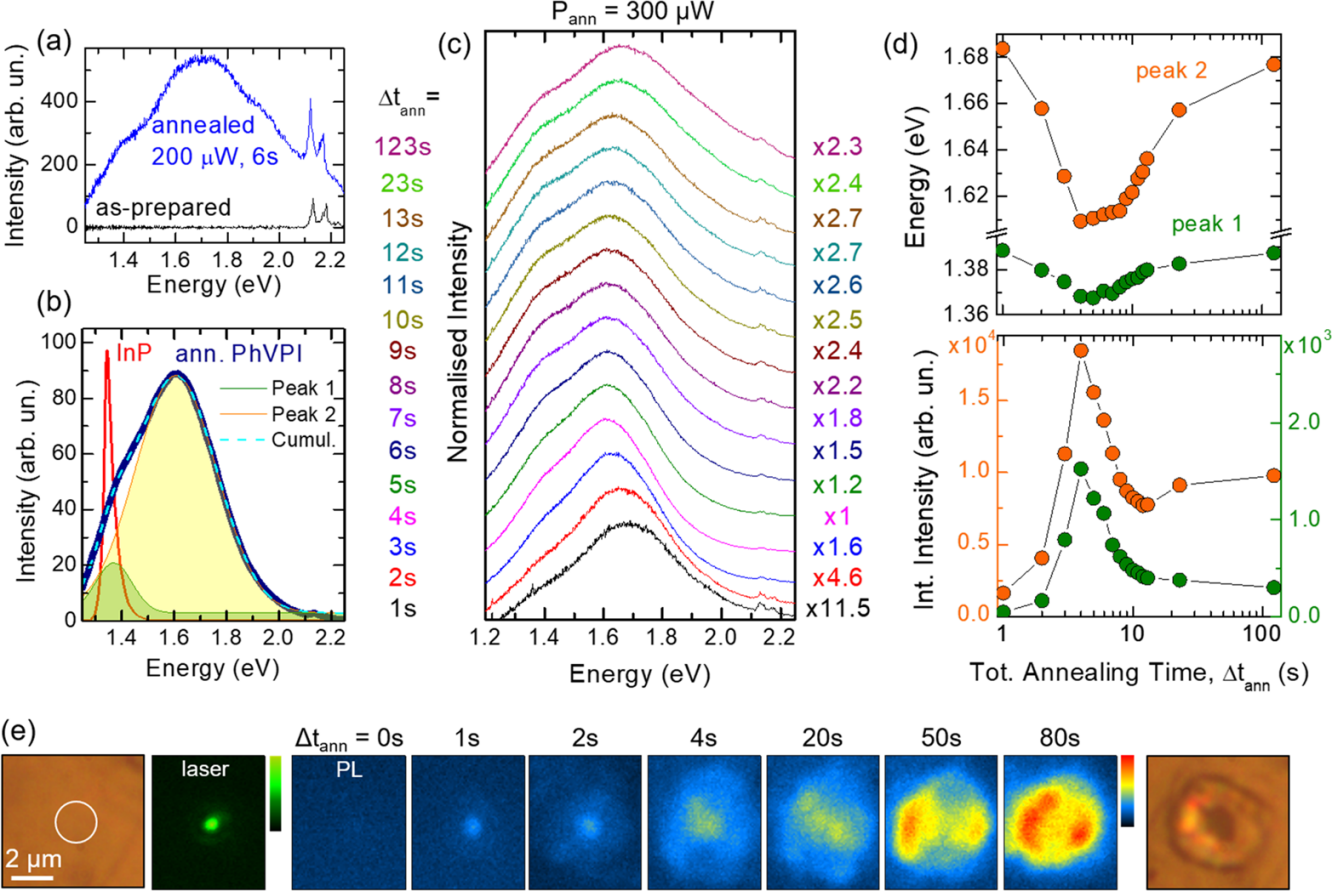
(a) PL spectrum (acquired with excitation power *P*_exc_ = 50 μW and with a 20× objective) of a PhVPI as-prepared sample (black), following annealing at *P*_ann_ = 200 μW for 6 s (blue). The spectrum of the as-prepared sample is dominated by the Raman peaks in the range 1000–1700 cm^–1^ (∼2.1 eV) associated with vibrational bending and stretching of the organics; see Figure S1 in the Supporting Information. However, no PL emission is visible. A PL signal appears instead after annealing. (b) Spectrum recorded after annealing at *P*_ann_ = 600 μW for 1 s and measured with *P*_exc_ = 0.12 μW, showing comparable peak-intensity to the PL band of an InP epilayer. The PhVPI spectrum was fitted with two gaussians. (c) Normalized PL spectra obtained by annealing a sample at *P*_ann_ = 300 μW for increasing cumulative annealing time Δ*t*_ann_, showing the line shape evolution upon annealing. A 20× objective was used. Information on the intensity evolution of the PL is given by the peak-intensity factors. The peak energy and integrated intensity of the two PL peaks—as obtained by the fits—vs Δt_ann_ is shown in panel d. (e) Spatial distribution of the PL intensity vs annealing time measured by projecting the signal onto a Si-CCD chip. A higher magnification objective (100×) was used for these studies. From left to right: Optical image of the sample, the laser being focused within the white circle; spatial distribution of the laser spot; spatial distribution of the PL signal for increasing Δ*t*_ann_; optical image of the sample at the end of the study. The annealing is performed by focusing the laser beam with *P*_ann_ = 300 μW via a 100× objective; the measurements are acquired with *P*_exc_ = 0.15 μW.

The PL band is dominated by two distinct transitions at ∼1.37 and ∼1.61 eV (see fits in Figure 2b). To follow the PL peak evolution vs annealing, we annealed a sample with *P*_ann_ = 300 μW using a 1 s annealing time, acquired the spectrum with *P*_exc_ = 50 μW, and we reiterated the same procedure several times; see Figures 2c-2d. An initial dramatic intensity increase upon annealing is observed for both the two transitions (Figure 2d, bottom). After a few seconds of annealing, however, an intensity decrease is observed, suggesting that a decomposition of the sample occurs for excessive annealing. This trend is accompanied by an initial redshift for both the two bands, followed by a blueshift when the intensity decreases (likely attributable to the sample decomposition at the center of the laser spot, with most of the PL signal coming from the surrounding less annealed regions); see Figure 2d, top. The initial redshift linked to the signal increase might originate from a local readjustment in the chemical bonds or by strain/distortion effects, related to the removal of residual solvent or by a crystal amorphization, as discussed below.

Analogous qualitative behaviors are found (i) for higher annealing powers (see Figure S2); (ii) by annealing the sample for a fixed annealing time of 5 min and increasing the annealing power (see Figure S3). Such a trend is also accompanied by changes in the Raman spectrum, revealing the appearance of new peaks for moderate annealing, followed by a signal quenching for excessive annealing; see Figure S4.

To better characterize the annealing effect, we studied the PL spatial distribution as a function of the annealing time, by projecting the signal onto a Si-CCD chip (see Supporting Information, Experimental Methods). An annealing power *P*_ann_ = 300 μW was employed, and a higher magnification objective (100×) was used to focus the laser and study the spatial distribution of the emitted light. In Figure 2e, from left to right, we show an optical image of the studied area and the spatial distribution of the laser spot. We then show the spatial distribution of the PL signal, and hence of the heat propagation in the sample. Indeed, no signal is observed prior to annealing. After 1 s of annealing, a signal can be observed in correspondence with the central part of the Gaussian laser spot. For longer annealing times up to ∼4 s the signal spreads, the brightest area still being at the center of the laser spot. For even longer annealing, the intensity at the center diminishes and the PL spatial distribution takes a ring-line shape, indicating that a decomposition is occurring at the center, as also supported by the change in morphology of the sample shown in the last panel.

The results obtained by laser annealing indicate a feasible route to turn the dark perovskite into an efficiently emitting material. In order to make this process scalable for potential applications, we performed thermal annealing studies in air (see Supporting Information, Methods) at temperatures equal to 100, 150, 200, 250, or 290 °C (i.e., below the PhVPI decomposition temperature = 332 °C^28^). No PL could be seen in samples treated at 200 °C or below for 1 h, while annealing for 1 h at 250 °C resulted in a bright PL emission. An even brighter signal was obtained by annealing at 290 °C for 5 min. The so-observed strong PL emission shows comparable intensity and line shape to that obtained by the best laser-annealing treatments; see Figure S5.

The structural evolution of PhVPI upon annealing was monitored by NMR and XRD (see Supporting Information, Methods). ^1^H NMR spectra showed no changes after annealing at 290 °C both in the phenyl viologen multiplets and in the organic cation signals. However, signals belonging to the solvent (DMF), from which the compound was formerly precipitated, were visible in the as-prepared sample, while disappearing after annealing, demonstrating the evaporation of the residual solvent.

The XRD pattern of the as-prepared sample (see Figure 3a), agrees with previous measurements,^28^ the material being completely crystalline. Analogous results were found in the sample annealed at 250 °C. The sample annealed at 290 °C shows instead a diffraction pattern with a substantial contribution of amorphous phase(s), reaching an amorphous content of 91 ± 1%.

**Figure 3:**
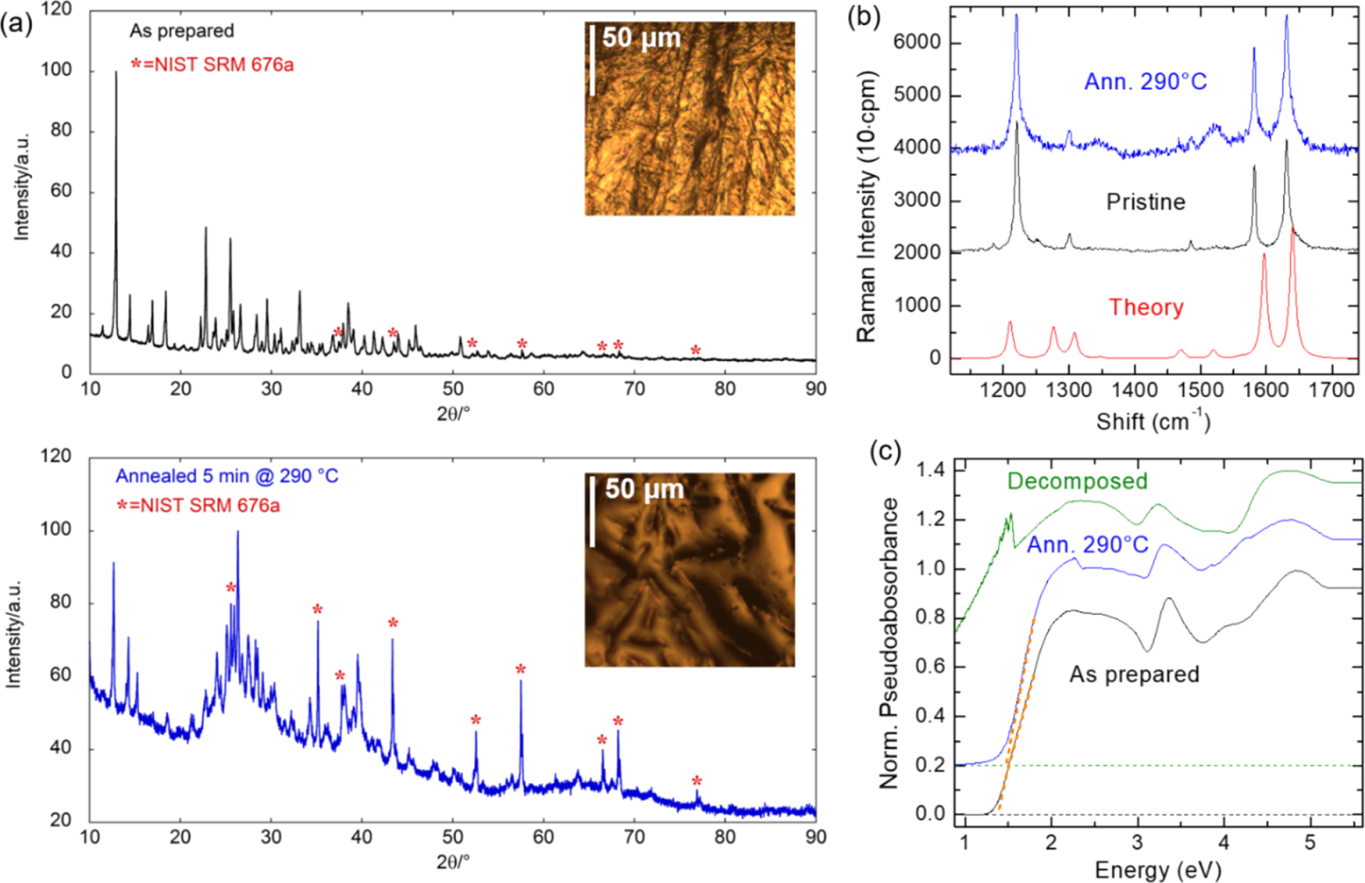
(a) X-ray diffraction spectra of the pristine and thermally annealed samples. Powder XRD patterns of the mixtures were performed with NIST SRM 676a (a Standard Reference Material for quantitative XRD made by the National Institute for Standards and Technology). While the as-prepared sample is completely crystalline, the sample annealed at 290 °C shows instead a diffraction pattern with a substantial contribution of amorphous phase(s) (i.e., a much lower signal-to noise ratio and more intense background contribution to the diffraction pattern). Rietveld QPA gives an amorphous content of 91 ± 1%. Insets: Optical images of the corresponding samples. (b) Corresponding Raman spectra in the middle frequency region. The theoretical spectrum is also shown for comparison. The data are stacked by *y* offset for clarity. (c) Normalized pseudoabsorbance spectra of the pristine, annealed and decomposed samples, stacked by a *y*-offset equal to 0.2 for clarity. The Tauc plots are also shown (see orange dashed lines). The annealed sample shows little variations with respect to the pristine one, consisting in slight shifts in the optical bandgap as determined by the Tauc plot, which shifts from 1.38 eV in the pristine sample to about 1.47 eV in the annealed sample. On the contrary, a dramatic variation can be observed in the decomposed sample, consistently with the PL quenching observed for excessive annealing.

Such an amorphization is also confirmed by modifications of the sample appearance (see Figure 3a). These findings, together with the NMR results—which exclude a chemical decomposition of PhVPI—indicate that the thermal treatments at 290 °C induce a substantial amorphization of PhVPI while preserving the crystal structure on a short-range scale, and thus without causing its decomposition, which occurs at 332 °C.^28^ This is also supported by Raman measurements (see Figure 3b), showing just a moderate increase/broadening of the peaks at 1350 and 1525 cm^–1^ (associated with vibrational bending and stretching of the organics; see Figure S1 in the Supporting Information).

To assess whether the annealing affects the optoelectronic properties of PhVPI, we measured the pseudoabsorbance spectrum (see Supporting Information, Methods) of both the pristine, annealed (at 290 °C), and decomposed (annealed at *T* > 332 °C) samples; see Figure 3c. The annealed sample shows little variations with respect to the pristine one, consisting in slight shifts, while a dramatic variation can be observed in the decomposed sample.

To gain further insight into the emissive properties of PhVPI, the variation of the PL emission vs temperature was investigated. Interestingly, the PL intensity and peak energy are unaffected when the lattice temperature *T* is decreased from room-temperature to 5 K (see Figure 4a), suggesting a strong localized character of the exciton wave function, which turns out to be of potential interest for room-temperature operating devices. In addition, we performed time-resolved PL measurements to measure the decay time associated with the two PL peaks; see Figure S6 in the Supporting Information. Our measurements show that (i) the two bands display decay times of few tens of picoseconds, and the low-energy band is characterized by lower decay times; (ii) *T* does not affect the decay times.

**Figure 4:**
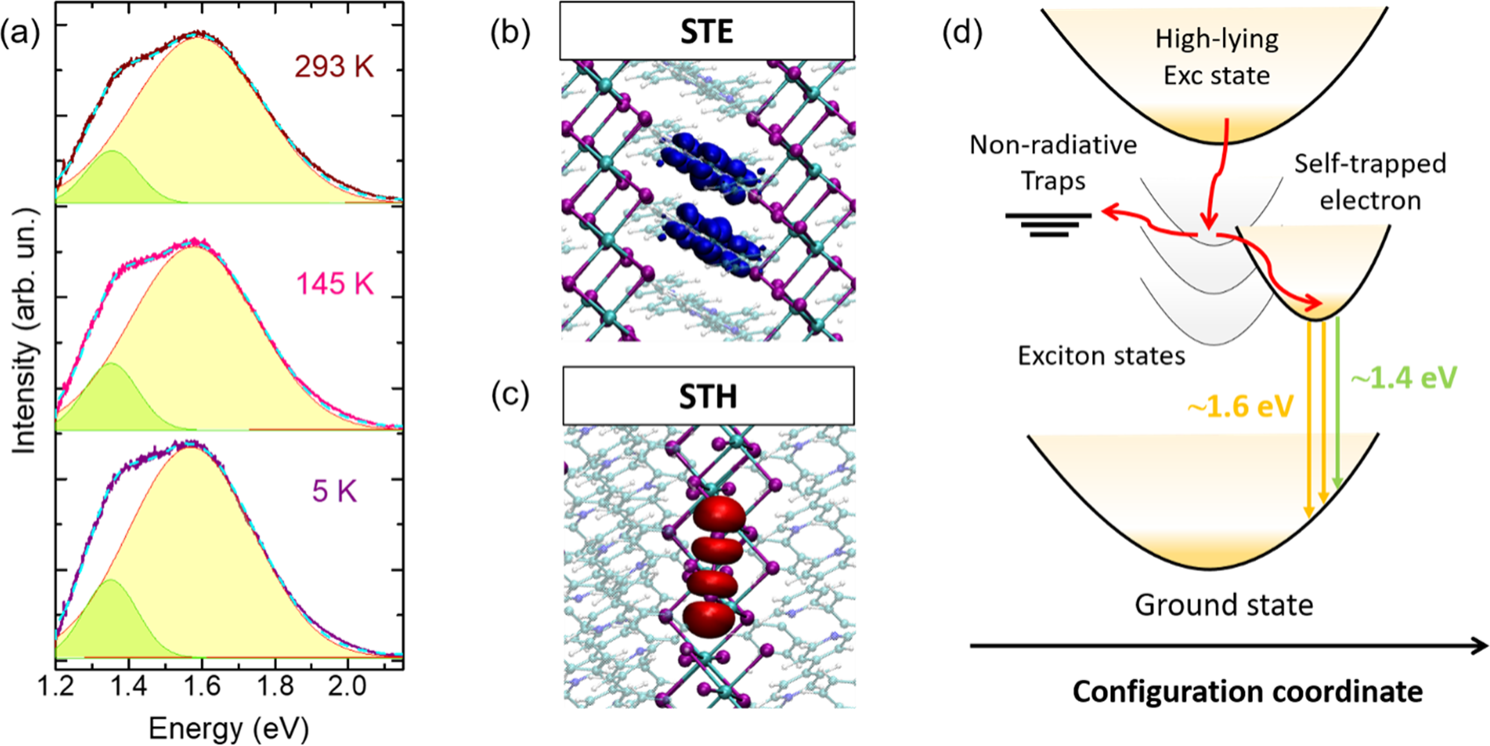
(a) PL spectra at room-temperature (293 K), 145 and 5 K; (b, c) Structures and isosurface orbital plots of the most stable configurations of (b) self-trapped electron (blue) and (c) self-trapped hole (red), i.e., the V_k_-center; (d) Diagram representing the emission processes in PhVPI. Transitions at ∼1.6 and ∼1.4 eV are ascribed to recombination of self-trapped electrons with holes in VB; nonradiative decay channels are associated with the formation of V_k_ centers.

The localization of excitons was investigated by DFT, simulating the self-trapping of excited charge-carriers in the pristine PhVPI within the supercell approach (see Supporting Information, Methods). Upon photoexcitation, the electron localizes on one single group of organic molecules in the supercell (see Figure 4b), being more stable than the delocalized electron by 0.26 eV. By simulating the recombination of the self-trapped electron (STE) with a delocalized hole in the VB, a PL emission at 1.71 eV is calculated, close to the PL peak at ∼1.61 eV. On the other hand, the most stable configuration of the self-trapped hole (STH) is associated with the formation of a V_k_ center, i.e., two iodines bound at ∼3.3 Å to form a I_2_^–^ species in the inorganic channel (see Figure 4c). The large reorganization energy accompanying the hole trapping process indicates that the formation of the V_k_ center is a potential non-radiative recombination channel.

These results indicate that the broad and thermally stable emission peaks at ∼1.61 and ∼1.37 eV mainly originate from the recombination of self-trapped electrons, following optical excitations; see Figure 4d. The similar nature of the processes originating these two transitions is further supported by the analogous behavior observed in PL power studies; see Figure S7. The relatively high stabilization energy of the electron upon self-trapping (0.26 eV) is compatible with the stable PL emission found at different temperatures. Optical activation by annealing is likely associated with removal of solvent molecules in the cavities—as also suggested by the NMR results—which can hinder electron relaxation in the organic moiety, by introducing alternative decay channels.

In conclusion, the optical properties of PhVPI were studied. This material shows a bright and thermally stable light emission after a short thermal annealing, comparable to that of highly efficient inorganic semiconductors, such as InP. Our study highlights that LHPs based on quaternary ammonium cations with an extended conjugation, like phenyl viologen, are excellent stable and tailorable perovskites. The enormous degree of freedom in the choice and functionalization of the cation may open infinite possibilities in the band gap tuning of this class of materials and may be exploited to obtain performant emissive devices in a wide spectral range.

## Supporting Information Available

Experimental and computational methods, laser annealing experiments results, comparison of emission intensity of PhVPI with InP, lifetime measurements results, power study of PL emission, additional experimental and computational results (PDF)
