# Supplementary material for: Brightly Luminescent and Moisture Tolerant Phenyl Viologen Lead Iodide Perovskites for Light Emission Applications
Source: J Phys Chem Lett. 2021 Jun 3;12(23):5456–62. doi: 10.1021/acs.jpclett.1c01271 (PMC8280716; doi:10.1021/acs.jpclett.1c01271)
Supplement: Supplementary file 1 — jz1c01271_si_001.pdf [file jz1c01271_si_001.pdf]

Supporting Information for

Brightly Luminescent and Moisture Tolerant Phenyl Viologen Lead Iodide Perovskites for Light Emission Applications

Elena Blundo[1], Antonio Polimeni*[1], Daniele Meggiolaro*[2], Andrea D'Annibale[3], Lorenza Romagnoli[3], Marco Felici[1], Alessandro Latini*[3]

[1]*Dipartimento di Fisica, Sapienza Università di Roma, Piazzale Aldo Moro 5, 00185 Roma, Italy*

[2]*Computational Laboratory for Hybrid/Organic Photovoltaics (CLHYO)Istituto CNR di Scienze e Tecnologie Chimiche "Giulio Natta"(CNR-SCITEC) Via Elce di Sotto 8, 06123 Perugia, Italy.*

[3]*Dipartimento di Chimica, Sapienza Università di Roma, Piazzale Aldo Moro 5, 00185 Roma, Italy*

email:

Antonio Polimeni: antonio.polimeni@roma1.infn.it

Daniele Meggiolaro: daniele.meggiolaro@cnr.it

Alessandro Latini: alessandro.latini@uniroma1.it

Methods and discussion

Synthesis. PhVPI was synthesized according to the procedure described in a previous work.[1] Thermal annealings were performed by treating the sample at 290 °C for 5 min. in air (ramp time from RT to 290 °C: 1 h).

X-ray diffraction. Powder X-ray diffraction analysis was performed by using a Malvern Panalytical X'Pert Pro MPD diffractometer equipped with a Ni-filtered Cu Kα source (λ=1.54184 Å) and an ultrafast RTMS X'Celerator detector. The determination of the amorphous content in the as prepared sample and in the thermally annealed sample was performed by the internal standard method[2] by mixing carefully weighted amounts of each sample with NIST 676a standard reference material (corundum phase Al_2O_3) and analysing the diffraction patterns of the mixtures with the Rietveld method for quantitative phase analysis (QPA) using the MAUD software package.[3]

NMR spectroscopy. ^{1}H NMR spectral analyses of as prepared and thermally annealed PhVPI were performed on samples dissolved in DMSO-d_6 using a 400 MHz Bruker Avance III spectrometer.
^{1}H-NMR spectra showed phenyl-viologen characteristic multiplets at δ 9.69 and 9.06 (assignable to protons of the pyridyl moiety in ortho and meta position relative to the aza group, respectively) in both the as-prepared and annealed sample (at 290 °C). The signals of the organic cation showed no changes after annealing. However, signals belonging to the solvent (DMF), from which the compound was formerly precipitated, were visible in the as-prepared sample, while disappearing after annealing, demonstrating the evaporation of the residual solvent.

Raman spectroscopy. For Raman measurements, the excitation laser was provided by a single frequency Nd:YVO_4 lasers (DPSS series by Lasos) with emission wavelength equal to 532.2 nm. The Raman signal was spectrally analysed by means of a 750-mm focal length monochromator Acton

SP750 equipped with a 1200 grooves/mm grating and detected by a back-illuminated Si CCD Camera (model 100BRX) by Princeton Instruments. The laser light was filtered out by a very sharp high-pass Razor edge filter (Semrock). The Raman spectral resolution was 0.7 cm^{-1}. A 20× objective (Olympus) with NA=0.4 was employed to excite and collect the light, in a backscattering configuration. The laser spot can be modelled as a gaussian with an experimentally measured σ = 0.75±0.02 μm.

Photoluminescence measurements. For photoluminescence (PL) measurements, the same experimental conditions used for Raman were employed (same excitation laser, same objective, backscattering configuration, Razor edge filter). In this case, the signal was spectrally analysed by means of a 200-mm focal length monochromator Isoplane 160 by Princeton Instruments, equipped with a 150 grooves/mm grating. The same CCD camera used for Raman was employed to detect the signal. The PL spectral resolution was 0.6 nm.

Photoluminescence spatial distribution. The PL spatial distribution was studied by projecting the PL signal onto a Si-CCD chip. This was done by performing measurements in a backscattering configuration, as discussed above. The collected signal was projected onto the chip by means of a long-focal-length lens and by keeping the slits opened. A long-working distance 100× objective (Zeiss, NA= 0.75) was used to excite the sample and collect the signal.

Time-resolved PL measurements. Time-resolved PL measurements were performed separately in the PL regions below 1.31 eV and above 1.91 eV. A supercontinuum laser at 530 nm was employed to excite the PL signal. An avalanche photodetector from MPD was employed to collect the signal.

UV-VIS spectroscopy. UV-VIS spectra in diffuse reflectance mode were acquired with a Shimadzu (Japan) UV2600 UV-Vis spectrophotometer equipped with an ISR-2600 Plus integrating sphere. $BaSO_4$ powder was used as reflectance standard.

Computational methods. Electronic and vibrational properties have been investigated by performing DFT calculations on the PhVPI unit cell (50 atoms) by fixing cell parameters to the experimental value, *i.e.* a=4.53 Å, b=12.61 Å, c=14.68 Å, α=107.9°, β=93.5°, γ=98.1°. All DFT calculations have been performed by using the Quantum Espresso code.[4]

Electronic bands and PDOS have been calculated by using the Perdew-Burke-Ernzherof (PBE)[5] functional and by including spin-orbit coupling (SOC). Single point PBE-SOC calculations have been performed on PhVPI optimized structures obtained by relaxing ion positions with the PBE functional and by including dispersion interactions through the DFT-D3 method.[6] Norm conserving pseudopotentials were used with a cutoff on the wavefunctions of 80 Ryd and a 8x2x2 grid of k-points in the BZ. Raman and infrared spectra of PhVPI have been simulated within the local density approximation (LDA)[7] by using norm-conserving pseudopotentials. A cutoff on the wavefunctions of 70 Ryd and a grid of 8x2x2 k-points in the BZ have been used.

G_0W_0 and BSE calculations were performed on the optimized PBE-DFT-D3 structure of PhVPI used in electronic structure calculations, by using the Yambo code.[8] G_0W_0 calculations have been carried out in the real axis integration approach by using 150 total energy steps, a cutoff of 30 Ry (3 Ry) for the exchange (correlation) part of the self-energy, and by including a total of 1024 bands in the calculation of the dielectric matrix and correlation energy. BSE calculations have been performed on top of QP corrected eigenvalues by using a cutoff of 30 Ry (3 Ry) on the exchange (screening) parts and by including 32 occupied and 28 unoccupied bands. A 6x2x2 grid of k-points in the BZ was used to reduce the computational effort. BSE spectrum has been calculated by averaging optical response in the three different x, y, z directions in space. Convergence tests on G_0W_0 band gap and BSE spectrum vs k-point grid are reported in Table S1 and Figure S9 of SI.

Self-trapping processes have been studied in the 4x2x2 supercell of PhVPI by using the CP2K package.[9] The hybrid PBE0 functional[10] was used by including dispersion interactions through the

the revised Vydrov-van Voorhis nonlocal van der Waals density functional.[11] Cell parameters were kept fixed at the experimental values. DZVP basis set[12] and norm-conserving Goedecker-Teter-Hutter pseudopotentials[13] have been used with a cutoff on the charge density of 300 Ry. The auxiliary density matrix method[14] has been used to accelerate hybrid functional calculations. Thermodynamic ionization levels (TIL) of self-trapped electrons and holes were calculated by using the following expression[15]

$$\varepsilon(q/q') = \frac{E[X^q] - E[X^{q'}]}{q' - q} + \frac{E_{corr}^{q} - E_{corr}^{q'}}{q' - q} - \varepsilon_{\mathrm{VB}}$$

Where $E[X^q]$ are the energies of self-trapped systems in the different state of charge q, ε_{VB} is the valence band of the pristine system and $E^q{}_{corr}$ are finite-size supercell corrections for charged defects. Makov-Payne corrections have been applied by using the static dielectric tensor of the perovskite ε_{xx}=8.6, ε_{yy}=6.6, ε_{zz}=5.7, estimated by following the approach of Umari *et al.*[16] PL emission energies have been calculated by simulating vertical transitions between the excited (trapped) and the ground state potential energy surfaces at the excited state equilibrium geometry.

Computational modelling of self-trapping processes in PhVPI

To investigate the nature of localized excitons, the self-trapping of excited charge carriers in the pristine PhVPI was simulated within the supercell approach. Calculations were performed in the 4×2×2 supercell of PhVPI by using the hybrid PBE0 functional[10] and neglecting SOC, due to its limited impact on the electronic properties of the perovskite. At this level of theory, a band gap of 2.07 eV is calculated, in good agreement with G_0W_0 calculations.

The self-trapping of the electron (hole) in the pristine perovskite was investigated by adding one extra negative (positive) charge and relaxing ions in the cell. Upon relaxation, the electron localizes on one single group of organic molecules in the supercell (see Figure 4b of the Manuscript) by leading to an increase of the dihedral angles in the central aromatic rings of the molecules from ~0° to a maximum

value of ~4°. The self-trapped electron (STE) is more stable than the delocalized electron by 0.26 eV and introduces a deep (0/-) thermodynamic ionization level (TIL) placed at 1.83 eV above the VBM. By simulating the recombination of the self-trapped electron with the delocalized hole in the VB, a PL emissions at 1.71 eV is calculated, close in energy to the broad PL emission peak measured at ~1.61 eV. The recombination of the STE with the hole in the same supercell by imposing the triplet state at the STE geometry leads to a redshifted emission at 1.46 eV, compatible with the less intense transition found at 1.37 eV in PL experiments. This red-shift is associated to the spontaneous localization of the hole in the inorganic chain proximate to the STE (see Figure S8). The localization of the hole in proximity of the STE increases the spatial overlap between the electron and the hole and their radiative recombination probability.[17]

On the other hand, the self-trapped hole (STH) shows two different minima. In the less stable configuration, the hole partially localizes on a single iodide row in the supercell by leading to a small lattice rearrangement and a relatively deep (+/0) TIL placed at 0.14 eV above the VBM. An optical emission from this semi-localized state at 1.76 eV is calculated, close to STE emission.

The most stable configuration of the self-trapped hole, however, is associated to the formation of a V_k center, i.e. two iodines bound at ~3.3 Å to form a I_2^- species in the inorganic channel (see Figure 4c of the Manuscript). This configuration is more stable than the delocalized hole by 0.53 eV and introduces a deep (+/0) TIL placed at 0.50 eV above the VBM. The large reorganization energy accompanying the hole trapping process indicates that the formation of the V_k center is a potential non-radiative recombination channel, even though a thermodynamic barrier to displace iodides from lattice sites and to form the V_k center is expected.

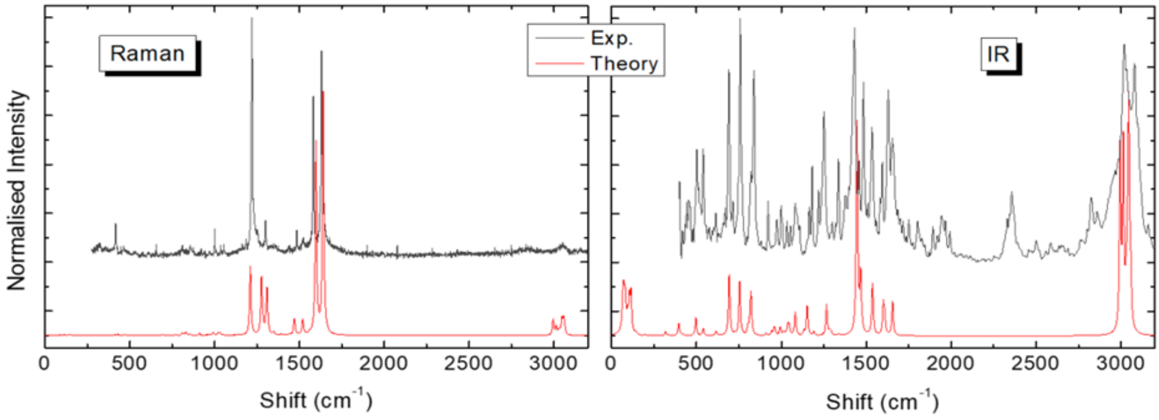

Figure S1. Comparison between the calculated (red) and experimental (dark grey) Raman (left) and IR (right) spectra. The experimental IR spectrum was taken from ref. 1 The Raman spectra were acquired by laser excitation at 532.2 nm in a confocal microscope setup, as detailed in the Methods section. Three distinct regions in the phonon spectra can be identified: i) a low frequency region < 150 cm^{-1} associated to bending and stretching of the inorganics partially coupled to the torsion of the organic cation; ii) an intermediate frequency range between 150-1700 cm^{-1} associated to torsion, bending and stretching of the organic cation rings; iii) a high frequency range around 3000 cm^{-1} associated to the C-H stretching in the organic cation. Notably, some peaks in the IR 1700-3000 cm^{-1} range of the experimental spectrum are absent in the simulated spectrum. We ascribe these peaks to solvent impurities in the as-synthesized sample.

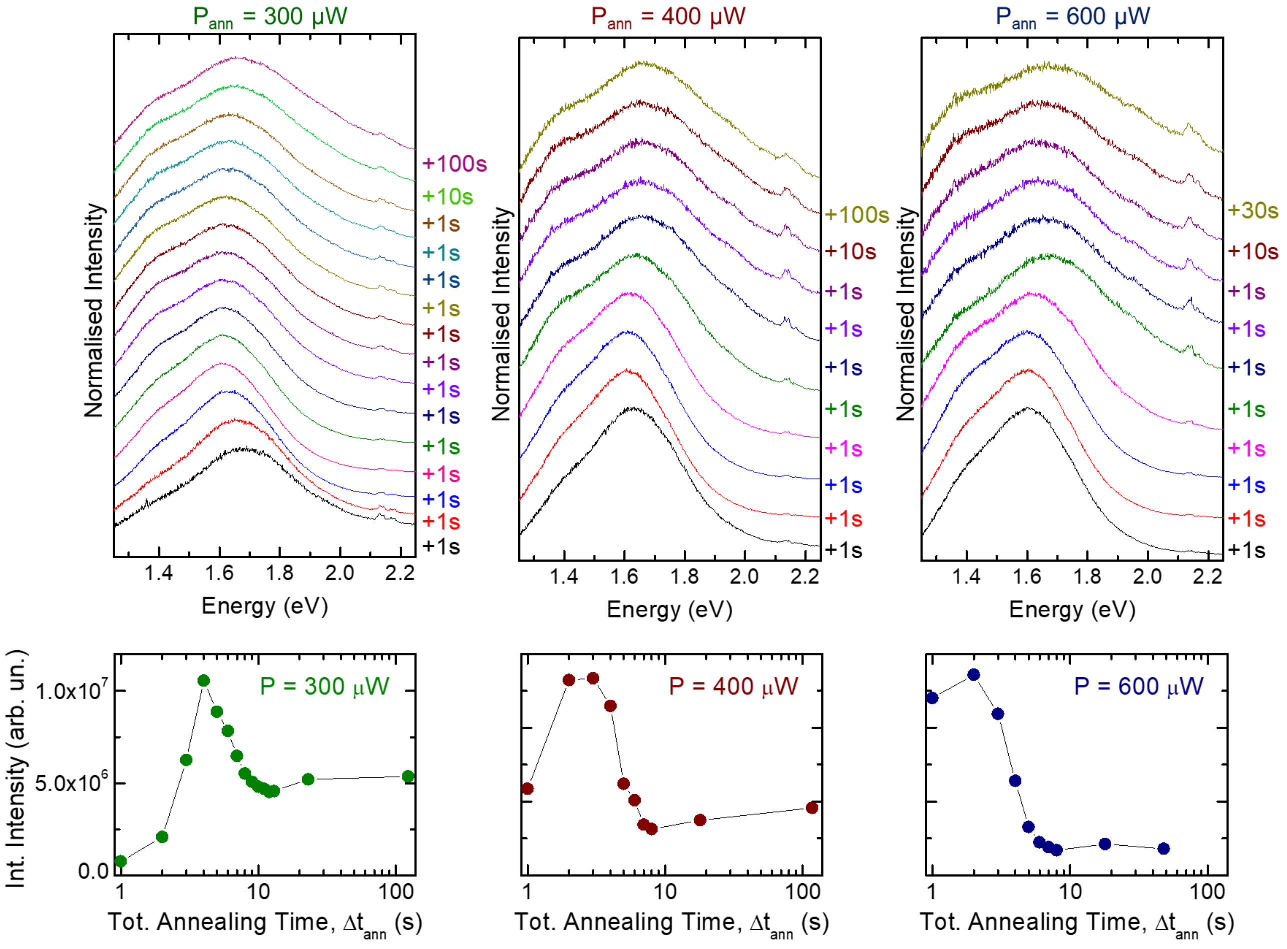

Figure S2. PL rise upon laser annealing in PhVPI. Top: Normalised spectra acquired with excitation power P_{exc} = 50 µW, as a function of the annealing time, and for three different annealing powers. Bottom: Integrated intensity behaviour as a function of the cumulative annealing time, obtained by the spectra shown above. Indeed, the same qualitative behaviour is observed in the three cases, with an initial rise of the PL intensity followed by its quenching. By increasing the annealing power, the maximum intensity is observed for shorter annealing times.

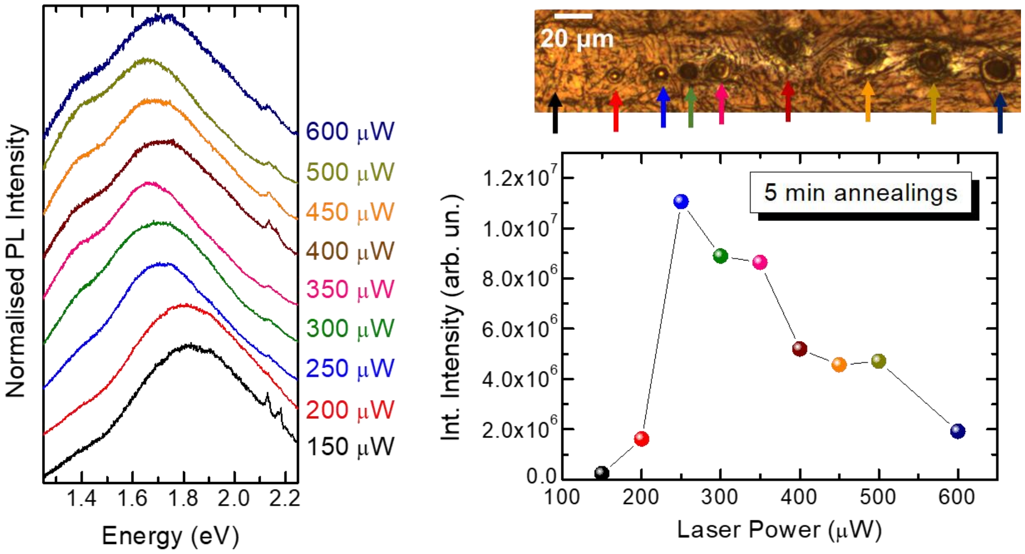

Figure S3. Laser annealing studies of the PhVPI, for 5-minute-annealings. Left: Normalised spectra acquired with excitation power P_{exc} = 50 μW, by annealing the sample for 5 minutes in different positions with different annealing times. Right: Optical image of the sample after the annealing study and integrated intensity of the spectra shown on the left, as a function of the laser annealing power.

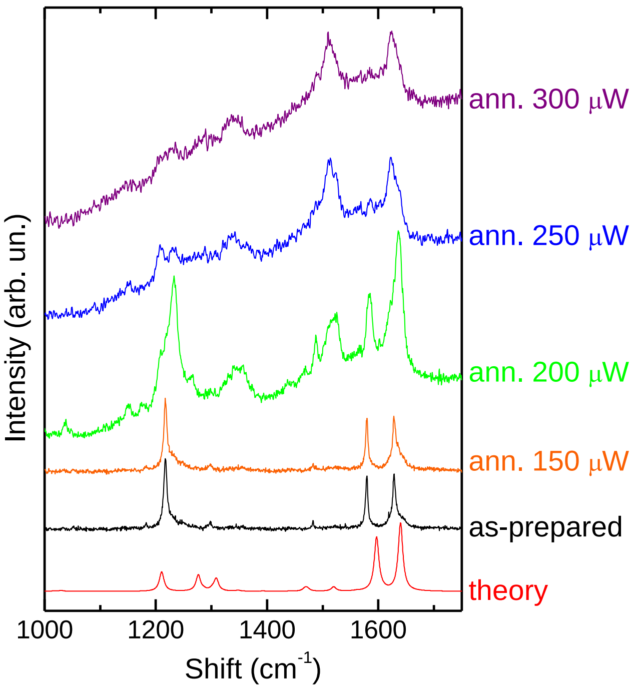

Figure S4. Raman studies of the annealed PhVPI. Raman spectra acquired with excitation power P_{exc} = 50 μW, after laser-annealing the sample for 1s with different annealing powers. The theoretical spectrum and that of the as-prepared sample are also shown for comparison. No changes can be seen up to annealing powers of about 150 μW, while extra peaks appear for higher powers, while the Raman intensity starts decreasing. The slope in the spectra is due to the queue of the PL emission.

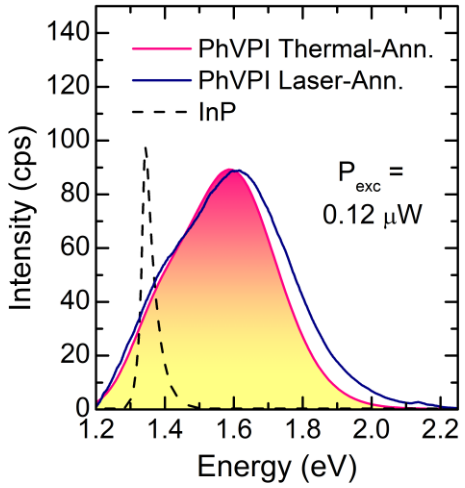

Figure S5. Comparison between the PL emission from the thermally-annealed and laser-annealed samples and InP. PL spectra acquired with P_{exc} = 0.12 μW in the most efficient thermally-annealed (annealed at 290 °C for 5 minutes) and laser-annealed samples we obtained. The spectra are compared to that of a high-quality InP epilayer, showing comparable peak intensity. Indeed, the PL spectra from the PhVPI are much broader, implying a much larger integrated intensity.

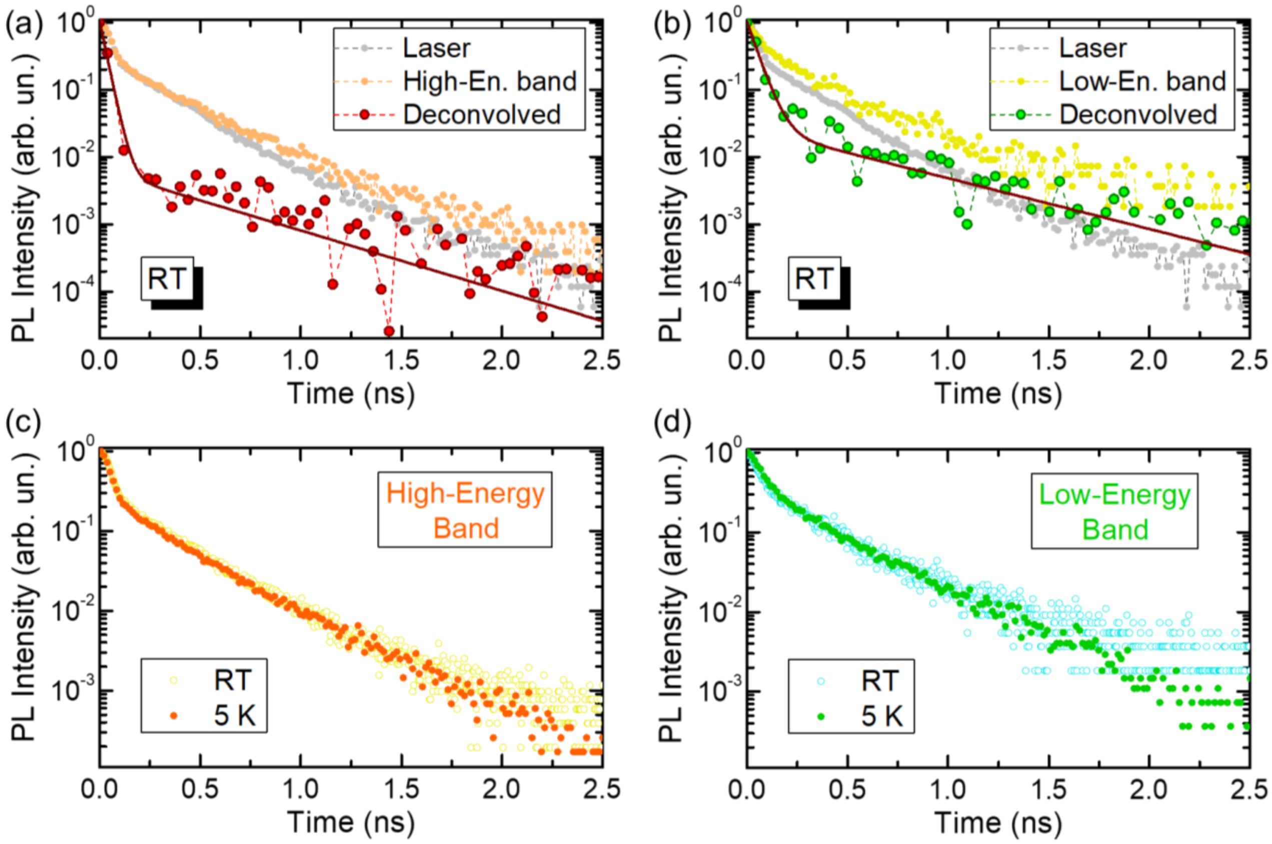

Figure S6. (a) Decay curves obtained via time-resolved PL measurements (see methods) performed at room-temperature (RT) on the high-energy PL band. The laser curve delimits the resolution limit of our setup. By performing a deconvolution procedure to subtract the system response from the signal curve, it is possible to estimate the decay times of the PL signal via a double exponential fit (solid line). The estimated lifetimes are $\tau_1 = (30.2 \pm 1.1)$ ps and $\tau_2 = (484 \pm 78)$ ps. (b) Same as panel (a) in the case of the low-energy PL band. In this case, longer lifetimes are estimated: $\tau_1 = (49.3 \pm 2.5)$ ps and $\tau_2 = (575 \pm 77)$ ps. (c) Comparison between the time-resolved PL spectra acquired at room temperature (RT) and at 5 K, on the high-energy band, showing an almost perfect overlap. (c) Same as panel (b) for the low-energy band. The differences observable towards ~2 ns are due to the lower signal-to-noise ratio at RT with respect to 5 K.

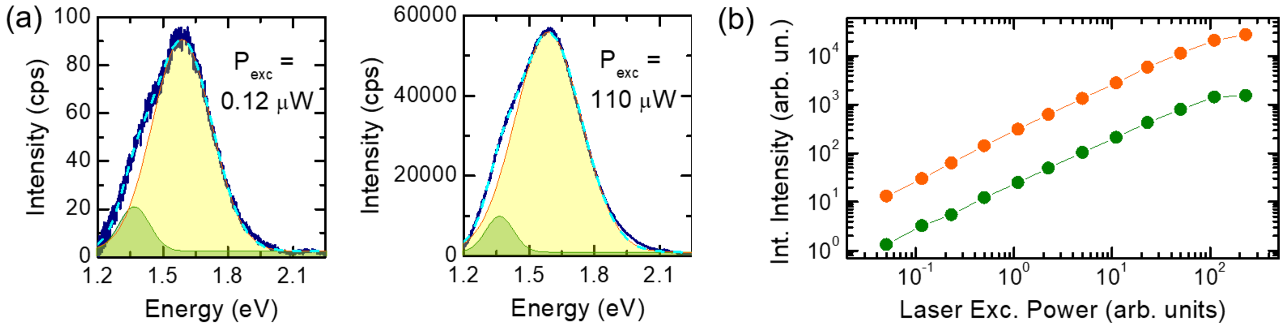

Figure S7. Power study of the PL emission. (a) Exemplifying PL spectra (dark blue) acquired in the thermally-annealed sample, with excitation powers differing by three orders of magnitude. The spectra were fitted with two gaussians (in green and orange), and the cumulative curves are also shown (cyan dashed line). A similar behaviour can be observed despite the quite different excitation power. (b) Excitation power dependence of the integrated intensity of the two PL bands, showing how they feature an analogous behaviour.

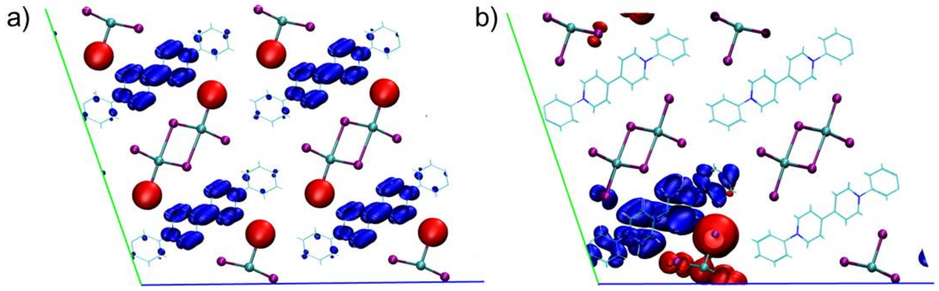

Figure S8. (a) Plot of the highest occupied molecular orbital (HOMO, in red) and the lowest unoccupied molecular orbitals (LUMO, in blue) of the pristine PhVPI perovskite, as calculated in the 4x2x2 supercell at the PBE0 level of theory. HOMO and LUMO are delocalized on the inorganic and organic moiety, respectively. (b) plot of the self-trapped electron (STE) orbital and the hole orbital in the triplet state simulated at the STE equilibrium geometry. The localization of the electron on a single group of organic cations promotes the localization of the hole in the proximate inorganic chain, by partially increasing their overlap in space.

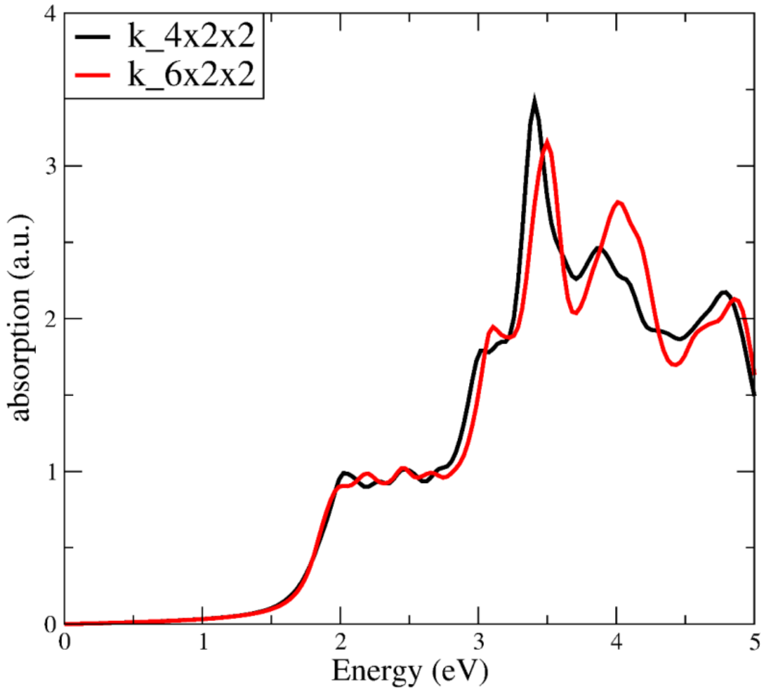

Figure S9. BSE spectra of PhVPI calculated on top of G_0W_0 corrected eigenvalues for different k-point grids in the BZ. The number of bands for the screening term has been reduced to 512, cutoff of 30 Ry (3 Ry) have been used for the exchange (screening) in the BSE hamiltonian. The spectra have been calculated as the optical response to electric field in the (1,1,1) direction in space by including 22 occupied and 18 unoccupied bands.

Table S1. Convergence of the direct energy gap at Γ vs computational parameters in G_0W_0 calculations. Convergence on the number of bands and cutoff on exchange (EXXRLvcs) and dielectric matrix (NGsBlkXd) have been carried out in the plasmon-pole approximation to reduce computational effort. In all cases the same number of bands have been used to calculate dielectric matrix and correlation energy. A smaller band-gap is obtained by using a real axis full frequency approach. Based on convergence tests the following computational setup for (EXXRLvcs / NGsBlkXd / number of bands / frequency steps) has been used (30 Ry, 3 Ry, 1024, 150).

**Computational setup**	**Direct energy gap at Γ (eV)**
**Plasmon Pole approximation, k grid 6x2x2**	
(EXXRLvcs / NGsBlkXd / number of bands)	
30 Ry, 3 Ry, 1024	2.43
40 Ry, 6 Ry, 1024	2.39
30 Ry, 3 Ry, 1536	2.43
30 Ry, 3 Ry, 2048	2.41
**Real axis frequency convergence, k grid 6x2x2**	
(EXXRLvcs / NGsBlkXd / number of bands / energy steps)	
30 Ry, 3 Ry, 1024, 100	2.31
30 Ry, 3 Ry, 1024, 150	2.06
30 Ry, 3 Ry, 1024, 200	2.08

References

(1) Latini, A.; Quaranta, S.; Menchini, F.; Lisi, N.; Di Girolamo, D.; Tarquini, O.; Colapietro, M.; Barba, L.; Demitri, N.; Cassetta, A. A Novel Water-Resistant and Thermally Stable Black Lead Halide Perovskite, Phenyl Viologen Lead Iodide $C_{22}H_{18}N_2(PbI_3)_2$. *Dalton Trans.* **2020**, 49, 2616-2627.
(2) Cline, J. P.; Von Dreele, R. B.; Winburn, R.; Stephens, P. W.; Filliben, J. J. Addressing the Amorphous Content Issue in Quantitative Phase Analysis: The Certification of Nist Standard Reference Material 676a. *Acta Crystallographica Section A* **2011**, 67, 357-367.
(3) Lutterotti, L.; Chateigner, D.; Ferrari, S.; Ricote, J. Texture, Residual Stress and Structural Analysis of Thin Films Using a Combined X-Ray Analysis. *Thin Solid Films* **2004**, 450, 34-41.
(4) Giannozzi, P.; Baroni, S.; Bonini, N.; Calandra, M.; Car, R.; Cavazzoni, C.; Ceresoli, D.; Chiarotti, G. L.; Coccioni, M.; Dabo, I., et al. Quantum Espresso: A Modular and Open-Source Software Project for Quantum Simulations of Materials. *Journal of Physics: Condensed Matter* **2009**, 21, 395502.
(5) Perdew, J. P.; Burke, K.; Ernzerhof, M. Generalized Gradient Approximation Made Simple. *Phys. Rev. Lett.* **1996**, 77, 3865-3868.
(6) Grimme, S.; Antony, J.; Ehrlich, S.; Krieg, H. A Consistent and Accurate Ab Initio Parametrization of Density Functional Dispersion Correction (Dft-D) for the 94 Elements H-Pu. *J. Chem. Phys.* **2010**, 132, 154104.
(7) Perdew, J. P.; Zunger, A. Self-Interaction Correction to Density-Functional Approximations for Many-Electron Systems. *Phys. Rev. B* **1981**, 23, 5048-5079.
(8) Marini, A.; Hogan, C.; Grüning, M.; Varsano, D. Yambo: An Ab Initio Tool for Excited State Calculations. *Comput. Phys. Commun.* **2009**, 180, 1392-1403.
(9) VandeVondele, J.; Krack, M.; Mohamed, F.; Parrinello, M.; Chassaing, T.; Hutter, J. Quickstep: Fast and Accurate Density Functional Calculations Using a Mixed Gaussian and Plane Waves Approach. *Comput. Phys. Commun.* **2005**, 167, 103-128.
(10) Adamo, C.; Barone, V. Toward Reliable Density Functional Methods without Adjustable Parameters: The Pbe0 Model. *J. Chem. Phys.* **1999**, 110, 6158-6170.
(11) Tran, F.; Hutter, J. Nonlocal Van Der Waals Functionals: The Case of Rare-Gas Dimers and Solids. *J. Chem. Phys.* **2013**, 138, 204103.
(12) VandeVondele, J.; Hutter, J. Gaussian Basis Sets for Accurate Calculations on Molecular Systems in Gas and Condensed Phases. *J. Chem. Phys.* **2007**, 127, 114105.
(13) Goedecker, S.; Teter, M.; Hutter, J. Separable Dual-Space Gaussian Pseudopotentials. *Phys. Rev. B* **1996**, 54, 1703-1710.
(14) Guidon, M.; Hutter, J.; VandeVondele, J. Auxiliary Density Matrix Methods for Hartree−Fock Exchange Calculations. *J. Chem. Theory Comput.* **2010**, 6, 2348-2364.
(15) Van de Walle, C. G.; Neugebauer, J. First-Principles Calculations for Defects and Impurities: Applications to III-Nitrides. *J. Appl. Phys.* **2004**, 95, 3851-3879.
(16) Umari, P.; Pasquarello, A. Ab Initio Molecular Dynamics in a Finite Homogeneous Electric Field. *Phys. Rev. Lett.* **2002**, 89, 157602.
(17) Wang, X.; Meng, W.; Liao, W.; Wang, J.; Xiong, R. G.; Yan, Y. Atomistic Mechanism of Broadband Emission in Metal Halide Perovskites. *J. Phys. Chem. Lett.* **2019**, 10, 501-506.
